# Role of the Phosphatase PTEN in Early Vascular Remodeling

**DOI:** 10.1371/journal.pone.0055445

**Published:** 2013-03-22

**Authors:** Daniel G. Sedding, Rebecca Widmer-Teske, Andreas Mueller, Philipp Stieger, Jan-Marcus Daniel, Dursun Gündüz, Soni Pullamsetti, Holger Nef, Helge Moellmann, Christian Troidl, Christian Hamm, Rüdiger Braun-Dullaeus

**Affiliations:** 1 Department of Internal Medicine I, Cardiology/Angiology, Giessen University, Giessen, Germany; 2 Division of Cardiology, University Hospital Magdeburg, Magdeburg, Germany; National Center for Scientific Research Demokritos, Greece

## Abstract

**Background:**

The phosphatase PTEN represents an important physiological inhibitor of phosphatidylinositol-3 kinase (PI3-K)/protein kinase B (Akt) signalling, however, the functional role of PTEN in the initial phase of angioplasty-induced vascular injury remains elusive. In the present study we sought to determine PTEN's effect on vascular smooth muscle cell (VSMC) apoptosis following acute injury *in vivo* and *in vitro*.

**Methods and Results:**

Immunohistochemistry indicated a faint basal expression and equal distribution of PTEN in uninjured rat carotid arteries. 12 h following balloon-injury, PTEN expression was strongly increased in apoptotic (TUNEL+) VSMC. In vitro, stimulation with serum or different growth factors or subjecting VSMC to cyclic stretch had no effect on PTEN expression, whereas stimulation with H_2_O_2_ robustly increased PTEN expression in a time- and dose-dependent manner. To evaluate the functional role of PTEN expression, human VSMC were transduced with WT-PTEN. Overexpression of PTEN increased the number of apoptotic VSMC (19.8%±4.4 vs. 5.6%±2.3; *P*<0.001) as determined by TUNEL assay. In contrast, siRNA-mediated knock-down of PTEN attenuated the basal as well as H_2_O_2_-induced apoptosis of VSMC. Mechanistically, overexpression of PTEN prevented serum-induced Akt-phosphorylation, whereas siRNA-mediated knock down of PTEN augmented Akt-activation. Moreover, co-transfection of PTEN and a constitutive active Akt mutant prevented PTEN-dependent augmentation of VSMC apoptosis, indicating, that PTEN regulates VSMC apoptosis by inhibition of Akt phosphorylation/activation.

**Conclusion:**

By interfering with the PI3-K/Akt-dependent survival signalling, the oxidative stress-induced up regulation of PTEN in VSMC of injured arteries augments the sensitivity of VSMC to apoptotic stimuli in the early phase following vascular injury, augmenting the initial injury and cell loss of the injured vessel wall. Thus, these data add to our understanding of PTEN's role during vascular remodelling.

## Introduction

Vascular proliferative disease such as postangioplasty restenosis is associated with multiple cellular processes such as inflammation, apoptosis and vascular proliferation [1]. Apoptotic death of vascular smooth muscle cells (VSMC) has been reported as an important component, especially in the early phase following vascular injury [2]. The mechanisms regulating apoptosis and cell proliferation, triggered by reactive oxygen species, have been intensively investigated, however, the complex interplay between apoptosis, cell proliferation, arterial remodeling and restenosis are not entirely elucidated so far.

The phosphatase and tensin homology deleted on chromosome 10 (PTEN), has been identified as a prominent tumor suppressor protein and important regulator of cell growth and apoptosis. PTEN acts as a dual-specificity lipid- and protein phosphatase and hydrolyzes the 3-phosphoinositide lipid products of PI3K thereby inhibiting the activation of a number of effector molecules downstream of PI3K [3], [4]. In a recent report, PDGF-induced proliferation and migration of VSMC as well as neointimal hyperplasia was successfully inhibited by PTEN-overexpression [5], [6]. Deletion of PTEN in all mouse tissues resulted in developmental delay and lethality at E 7.5 due to unbalanced tissue growth during early embryogenesis and subsequent organ failure [7]. Recently, using tamoxifen-inducible SMC-specific PTEN knockout mice, it was documented that inactivation of PTEN *in vivo* exacerbates injury-induced neointima formation [8].

However, the role of PTEN during the initial phase following vascular injury and its impact on cell survival remains obscure. In this study we show for the first time that PTEN expression is upregulated in VSMC following vascular injury *in vivo* and *in vitro*, triggered by oxidative stress. Up regulation of PTEN renders VSMC more susceptible to basal and H_2_O_2_-induced apoptosis by interfering with Akt-dependent survival signaling. Thus PTEN augments initial cell loss and wall damage following vascular injury.

## Methods

### Ethics statement

All procedures involving experimental animals were approved by the institutional committee for animal research of the Giessen University (UGI 2002-6) and complied with the Guide for the Care and Use of Laboratory Animals (NIH publication No. 86-23, revised 1985).

### Balloon angioplasty of the rat common carotid artery

Adult male Wistar rats (300 g body weight, Harlan Winkelmann, Borchem, Germany) were anesthesized (35 mg/kg ketamine, Inresa, Freiburg, Germany; 5 mg/kg xylazine, AstraZeneca, Wedel, Germany), and the common carotid artery was isolated. A Fogarty 2F embolectomy catheter (Edwards Lifesciences, Unterschleissheim, Germany) was introduced into the common carotid artery through the external carotid branch, advanced, inflated, and withdrawn three times. Finally, the balloon catheter was removed, and the proximal external carotid branch suture was tied using a 4-0 silk ligature (Johnson & Johnson, Brussels, Belgium). Wounds were closed and buprenorphine (0.5 mg/kg, Essex Pharma, Munich, Germany) was given for analgesia. On full recovery, animals were returned to the animal care facility and provided standard rat chow and water *ad libitum*. At the indicated time points, the animals were euthanized by an isofluran overdose (Baxter, Unterschleissheim, Germany).

### Vessel Harvesting

For immunohistochemical evaluation, perfusion-fixation with 4% paraformaldehyde following removal, arteries were embedded in Tissue Tek OCT (Miles Laboratories, Naperville, IL), snap-frozen, and stored at −80°C until use. Samples were sectioned on a Leica cryostat (6 µm) and placed on poly-L-lysine (Sigma)–coated slides for immunohistochemical analysis. All sections were examined under a Leica DMRB microscope (Wetzlar, Germany). For protein isolation, carotid arteries were removed and lysed in RIPA buffer containing PBS, 1% NP-40, 0.5% sodium deoxycholate, 0.1% SDS, 10 µg/mL PMSF, 30 µg/mL aprotinin, 1 mol/L sodium orthovanadate as previously described [9].

### Stretching apparatus, and experimental conditions

Primary VSMC were isolated, cultured and transferred to the experiment as previously described [10], [11]. In brief, fresh serum-free medium was substituted and cyclic uniform uniaxial stretching was applied with a flexercell apparatus (FX-3000; Flexcell; 125% resting length, 0.5 Hz) in a tissue culture incubator.

### Immunohistochemistry

Cross sections of rat carotid arteries were fixed for 10 min in 4% paraformaldehyde. Sections were blocked with 10% normal goat serum, and incubated with anti-PTEN (1∶100, Cell Signaling, Beverly, MA) for 1 h in phosphate-buffered saline (PBS) containing 0.1% bovine serum albumin. After two washing steps, cross sections were incubated for 40 min with donkey anti-rabbit IgG conjugated to Alexa Fluor® 488 (1∶200, Molecular Probes, Leyden, The Netherlands). For detection of apoptotic cells, the terminal deoxynucleotidyl transferase-mediated dUTP-biotin nick end labeling (*TUNEL*) was employed according to the manufacturer's instructions (In situ cell death detection kit, Roche Diagnostics, Mannheim, Germany). After washing, slides were mounted in Vectashield® mounting medium H-1000 containing DAPI (5 µg/ml, Linaris, Wertheim, Germany) and evaluated using an epifluorescence microscope.

### Cell culture

Human coronary artery smooth muscle cells (HcASMC; Cambrex, Verviers, Belgium) were cultured in an incubator at 37°C and 5% CO_2_ using Smooth Muscle Cell Growth Medium 2 (PromoCell, Heidelberg, Germany) supplemented with penicillin (10'000 U/ml) and streptomycin (10'000 µg/ml). The cells were used until passage 6.

### Co-transfection and magnet-activated cell sorting of transfected cells

Transfection was performed using the cationic lipid-reagent “Fugene” (Roche Diagnostics, Mannheim, Germany) by incubating cells with plasmids containing WT-PTEN-cDNA or Akt-cDNA carrying a constitutively active form of Akt (both kindly provided by Kenneth Walsh, Boston, USA) together with the pMACS KK^II^ plasmid (1∶3) (Miltenyi Biotec, Auburn, CA, USA) to allow the selection of positively transfected cells as previously described (3-deaza circ res paper)). Briefly, cells were incubated in 35 mm dishes for 4 hours at 37°C with the plasmids in serum-free medium. Afterwards, the medium was replaced by medium containing 5% FCS. The cells were labeled with MACSselect KK^II^ microbeads (Miltenyi Biotec, Bergisch Gladbach, Germany) 24 hours later and selected by MACSselect KK^II^ column (Miltenyi Biotec) according to the manufacturer's protocol. Up to 85% of the cells were positively selected for K^k^-II expression. HcASMC were transfected as follows: non transfected (NT); transfected with a plasmid coding for GFP (pGFP); transfected with an empty plasmid without PTEN-cDNA (pControl); transfected with a plasmid coding for PTEN (pPTEN); mock transfected (without plasmid, but with transfection reagent).

### RNA interference

HcASMC at ∼50% confluency seeded in 6-well plates were transiently transfected with the indicated siRNA duplex by using Lipofectamine 2000 (Invitrogen GmbH, Karlsruhe, Germany) according to the manufacturer's instructions. In brief, diluted siRNA was combined with diluted Lipofectamine 2000 (1∶100), subsequently mixed with growth medium (without antibiotics) and added to the cells at a final concentration of 30 nM. Cells were grown for 24 h prior to experiments. Transfection of siRNA and controls were performed with each experiment as follows: non transfected (NT); mock transfected (without siRNA, but with lipid carrier); transfected with a non-targeting (scrambled) siRNA (Control siRNA) for detecting off-target effects; transfected with a targeting siRNA against PTEN (PTEN siRNA).

### Experimental setup

Prior to the *in vitro* experiments, transfected or non-transfected HcASMC were silenced in basal medium (Smooth Muscle Cell Basal Medium 2; PromoCell) for 48 h. Dependent on the experiment, cells were then either incubated in Smooth Muscle Cell Basal Medium 2 (PromoCell) with or without 500 µM H_2_O_2_ and with or without supplementation of the PTEN inhibitor potassium bisperoxo(bipyridine)oxovanadate (bpV, 200 nM), and in growth medium containing 10% FCS with or without the phosphoinositide 3-kinase (PI3K) inhibitor Ly294002 (50 µM) for 24 h.

### Phosphatase assay

For the determination of PTEN activity, PTEN protein either from dilated or undilated carotid arteries or cultured HcASMC following lysis was immunoprecipitated using an anti-PTEN antibody (Santa Cruz Laboratories, USA). Antibody-coupled, magnetic beads were employed for specific protein isolation following the manufacturer's instructions (Miltenyi Biotec, Bergisch Gladbach, Germany). Either an IgG-iso-antibody targeting no specific protein or no addition of any antibody served as controls. The reactions were incubated in phosphatase assay buffer containing 100 mM TrisHCl pH 8, 10 mM DTT, and 200 mM water-soluble diC8-PIP3 (Echelon, Salt Lake City, USA) for 40 min at 37°C and transferred to a 96-well plate. The release of phosphate from the substrate was measured in a colorimetric assay by using the Biomol Green Reagent (Biomol, Hamburg, Germany) in accordance with the instructions of the manufacturer. The absorbance at 650 nm was recorded in an ELISA plate reader. A standard curve was performed in each assay, and the amount of free phosphate was calculated from the standard curve line-fit data.

### Preparation of Cellular Lysates and Immunoblot Analysis

Semiquantitative analysis of proteins in cell lysates was performed by western blotting and antibody detection as previously described [9]. Briefly, the cleared supernatant from lysates was resolved on a polyacrylamide gel and blotted onto nitrocellulose (Hybond-ECL, Amersham, Freiburg, Germany) by wet electroblotting. After blocking, membranes were incubated with the following antibodies: anti-pan-Akt and p-Akt: 1∶1000; anti-PTEN: 1∶500; anti-p53: 1∶2000; anti-Cdk4: 1∶2000 (all from Santa Cruz Biotechnology, Santa Cruz, USA) for 1 h at room temperature. Proteins were then visualized by enhanced chemiluminescence (ECL+, Amersham) after labeling with horseradish peroxidase-labeled secondary antibody (1∶2000 for 1 h) according to the manufacturer's instructions. For the detection of PTEN oxidation, protein samples were isolated and separated under non-reducing conditions prior to immunoblotting as previously described[12].

### Quantification of HcASMC Apoptosis

Subconfluent (∼80%) HcASMC were cultured on chamber slides. Cells were fixed using 2% paraformaldehyde and terminal deoxynucleotidyl transferase-mediated dUTP nick end-labeling (TUNEL) was performed according to the supplier's instructions (In situ cell death detection kit, Roche Diagnostics, Mannheim, Germany). After nuclear staining with 4,6-Diamidino-2-phenylindole, 2HCl (DAPI), samples were viewed with an inverted fluorescence microscope (DMRB, Leica, Wetzlar, Germany) and two independent investigators, blinded to the treatment, evaluated the relative number of apoptotic cells per well by counting 4 randomly selected high-power fields.

For other experiments, apoptotic cells were analyzed using the Cell Death Detection ELISA Plus (Roche Diagnostics, Mannheim, Germany). The assay was performed according to the manufacturer's instructions. In brief, cells grown in 96-well plates were lysed, transferred into the microtiter plate provided with the assay and incubated in freshly prepared immunoreagent. The solution was removed, the wells rinsed with the provided incubation buffer and incubated in ABTS solution on a plate shaker until color development was sufficient for photometric analysis. Measurement was done at 405 nm against ABTS solution as a blank, with a reference wavelength of 490 nm.

### Quantification of cell proliferation

The quantification of cell proliferation was determined by measuring the incorporation of the pyrimidine analogue 5-bromo-2′-deoxyuridine (BrdU) into the genomic DNA of proliferating cells using the Cell Proliferation ELISA from Roche (Roche Diagnostics, Mannheim, Germany). In brief, cells grown in 96-well plates were incubated with BrdU labeling solution for the last 6 h of their cultivation and fixed with FixDenat. The cells were then incubated with anti-BrdU-POD antibody. After removal of the antibody conjugate, the cells were rinsed, substrate solution was added and incubated at room temperature until color development was sufficient for photometric detection. After stopping the reaction using H_2_SO_4_, the absorbance was measured in an ELISA reader at 450 nm against blank measurements (BrdU-medium), with a reference wavelength of 620 nm.

### Statistical Analyses

Data were stored and analyzed on personal computers using Excel 2007 (Microsoft) and Sigma Plot 8.0 with Sigma Stat 2.03 (Systat, Erkrath, Germany). Differences between the study groups were analyzed by ANOVA followed by a post hoc analysis using the Holm Sidak Test. All data are represented as mean ± standard deviation (SD). A probability value <0.05 was considered statistically significant for all comparisons.

## Results

### PTEN is upregulated in VSMC following vascular injury *in vivo*

**Figure 1:**
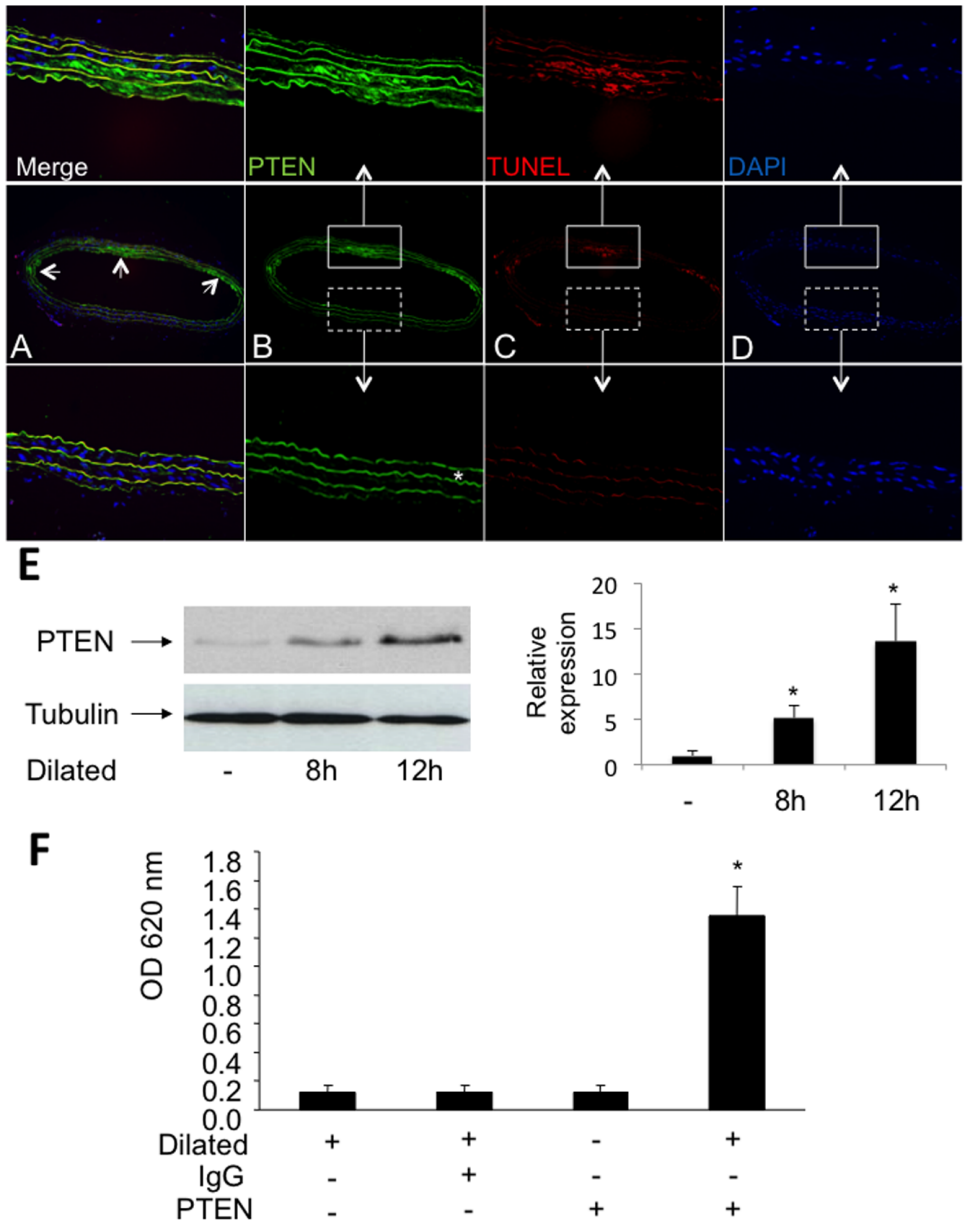
PTEN expression and apoptotic cells *in vivo*. **A–D**, Immunoreactivity of PTEN (green), apoptotic smooth muscle cells (TUNEL staining, red) and nuclei (DAPI, blue) is shown in representative sections of an injured rat carotid artery 12 h after balloon injury. The autoflourescence of the elastic laminae (* in figure 1B) allows the identification of the media. Strongly injured areas of the dilated vessel were identified by a reduced cellularity and by the accumulation of apoptotic cells. Higher magnifications of vessel areas which fulfill these criteria are shown in the upper panel whereas higher magnifications of less injured vessel areas are shown in the lower panel. **E** PTEN expression is upregulated in dilated rat carotid arteries *in vivo* as determined by western blotting using a specific PTEN antibody. Detection of β-tubulin served as loading control. The relative expression of PTEN as determined by densitometric analysis of immunoblots is shown (n = 3; *P<0.05). **F**, The activity of PTEN is enhanced after vascular injury. Shown is the phosphatase activity as determined by immunoprecipitated PTEN from lysates of dilated and undilated rat carotid arteries. Immunoprecipitations employing an IgG iso-antibody and without addition of any antibodies served as controls. Results are expressed as mean OD650 ± SD (*P<0.001, n = 4).

To investigate the effect of balloon injury on PTEN expression and apoptosis of SMC in rat carotid arteries, we conducted immunohistochemical analysis on frozen vessel sections 12 h after balloon dilatation. Within the area of severe injury and especially in apoptotic medial VSMC, which can be distinguished from the intact vessel area due to the lack of intact DAPI-stained nuclei in the damaged region, we detected a strong expression of PTEN (green) in apoptotic (TUNEL-stained; red) cells (Fig. 1A and B+C and Figure S1A–1C). The enhanced PTEN expression after vascular injury was also confirmed by immunoblotting, comparing protein expression from lysates of dilated and undilated vessels (Fig. 1E). Additionally, we analyzed whether vessel dilatation has also an effect on PTEN activity. By performing a phosphatase assay with PTEN immunoprecipitated from three pooled carotid arteries we could show that PTEN activity from dilated vessels was enhanced compared to undilated arteries (OD 1.35±0.2 vs. 0.125±0.05; *P<0.001; Fig. 1F). Immunoprecipitations employing an IgG iso-antibody and immunoprecipitations without addition of an antibody served as controls.

### H_2_O_2_ but not mitogenic stimulation or cyclic strain augments PTEN expression and activity *in vitro*

**Figure 2:**
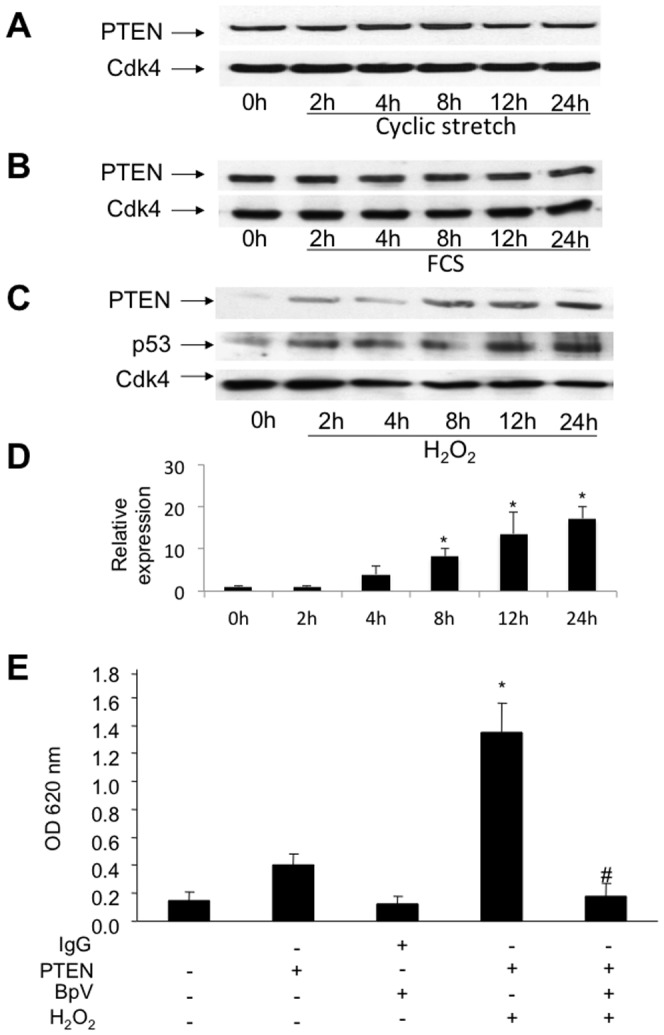
PTEN expression in human coronary VSMC *in vitro*. **A**, PTEN-expression is not upregulated by mechanical stress in VSMC. Lysates of cells exposed to mechanical forces using a stretching device were analyzed by western blotting using specific antibodies. **B**, Lysates of SMC exposed to growth medium (FCS). PTEN expression was not upregulated after 24 h. Protein expression was determined using specific antibodies. Detection of Cdk4 served as loading control. **C**, Lysates of SMC exposed to oxidative stress using 500 µM H_2_O_2_. PTEN upregulation was triggered by oxidative stress within 24 h. Detection of p53 and Cdk4 served as apoptotic marker and loading control, respectively. **D**, The upregulation of PTEN protein levels was quantified by densitometric analysis of immunoblots (n = 3; *P<0.05). **E**, The upregulation of PTEN activity is mediated by H_2_O_2_-induction. Shown is a phosphatase activity assay of immunoprecipitated protein from lysates of HcASMC with and without 24 h H_2_O_2_–treatment. Immunoprecipitations from lysates employing an IgG iso-antibody without H_2_O_2_-treatment with and without bpV supplementation, an anti-PTEN-antibody without H_2_O_2_ and bpV treatment and an anti-PTEN-antibody with H_2_O_2_– and bpV-treatment served as controls. Results are expressed as mean OD650 ± SD using an ELISA plate reader (^#^ *P*<0.001, *P<0.001; n = 4).

Since signaling pathways are regulated by reactive oxygen species and growth factors in vascular cells [1], [13] and apoptosis is induced by mechanic strain stress during PCI [10], we determined whether cyclic strain, serum stimulation and/or H_2_O_2_ result in PTEN-upregulation in SMC *in vitro*. Expression patterns were analyzed by immunoblotting of PTEN isolated from cultured SMC after exposure to mechanic strain in a stretching device (Fig. 2A), stimulation with 10% FCS (Fig. 2B) or 500 µM H_2_O_2_ for up to 24 h (Fig. 2C) following 48 h serum starvation. H_2_O_2_ rather than FCS or cyclic strain was found to augment PTEN-expression in a time-dependent manner suggesting that at least *in vitro* the exposure of SMC to oxidative stress results in a robust increase of PTEN expression. Concomitant upregulation of p53, a prominent marker for apoptosis, indicates that PTEN expression is accompanied by programmed vascular cell death following the exposure to oxidative stress. Detection of Cdk4 served as a loading control. Moreover, the addition of catalase prevented the H2O2-induced upregulation of PTEN, indicating that the enhanced expression is indeed triggered by reactive oxygen species (Figure S2). A phosphatase assay with immunoprecipitated PTEN from cultured SMC indicated that it's activity was strongly induced after 24 h H_2_O_2_ treatment as compared to untreated cells (OD 1.35±0.2 vs. 0.4±0.08; *P<0.001; Fig. 2D). The increase of PTEN-activity could be completely prevented by preincubation with the specific PTEN-inhibitor bpV at a concentration of 200 nM, indicating, that the measured activity indeed resembles PTEN-phosphatase-activity (OD 1.35±0.2 vs. 0.175±0.09; *^#^P*<0.001; Fig. 2D). To determine the effect of H_2_O_2_ on PTEN-oxidation, VSMC were exposed to 500 µM H_2_O_2_. Following 15 min of H_2_O_2_ incubation, almost all of the cellular PTEN protein-content was oxidized as indicated by a band shift in a non-reducing gel. In contrast, incubation of VSMC with H_2_O_2_ for 12 h increased total PTEN protein content but did not result in a prominent oxidation (band shift) of the protein (Fig. S2B). Moreover, PTEN expression in dilated arteries 12 h following injury was robustly increased, but no significant oxidation of the protein (band shift) was detected by immunoblotting under non-reducing conditions. These results indicate, that PTEN is not inactivated by oxidation at 12 h following vascular injury *in vivo*, and that the enhanced enzymatic activity is most likely due to it's increased protein content.

### PTEN is a critical regulator of H_2_O_2_-induced apoptosis of VSMC

**Figure 3:**
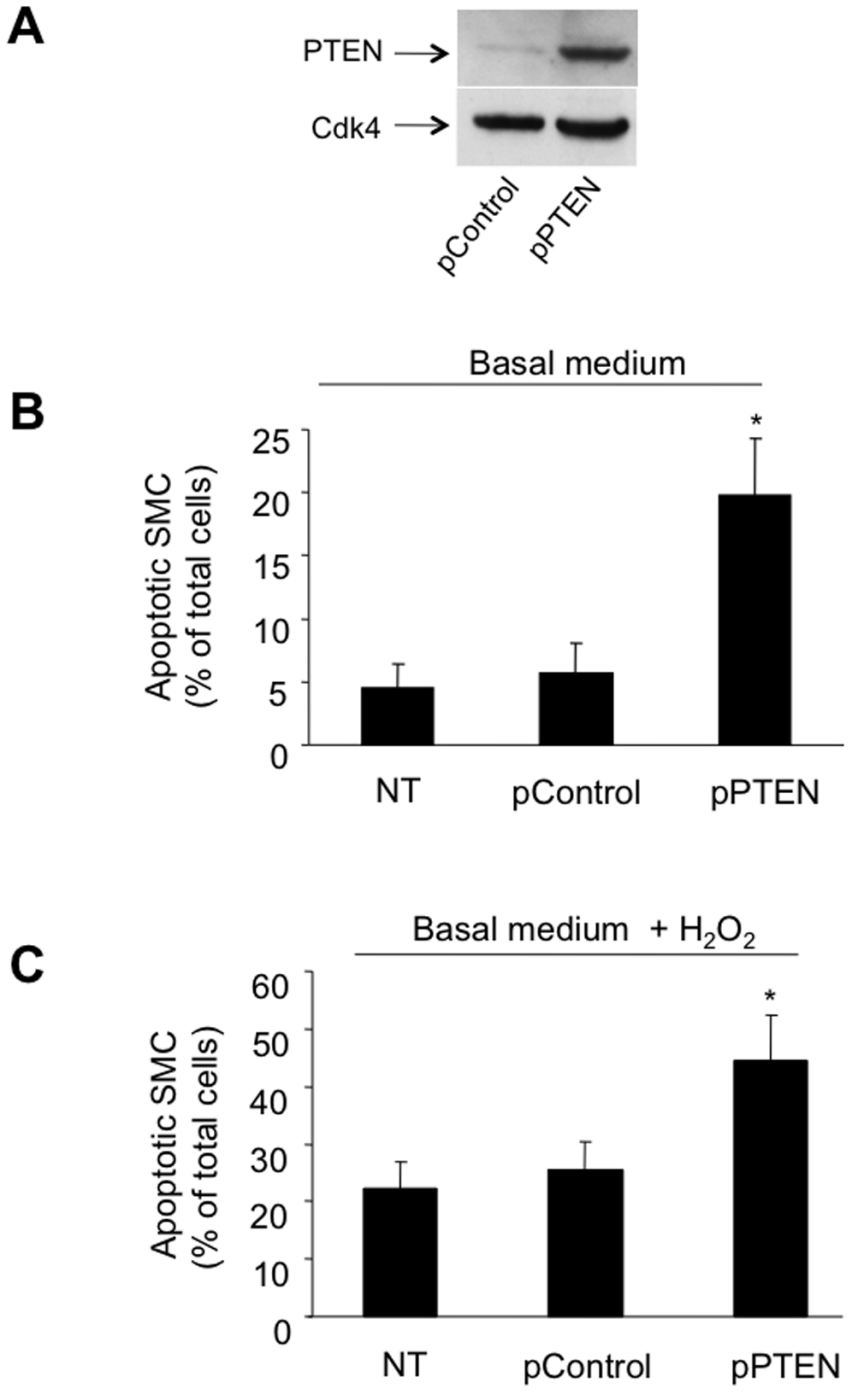
PTEN overexpression augments SMC apoptosis under basal conditions as well as following exposure to oxidative stress. HcASMC transfected with a plasmid carrying WT-PTEN or a control (empty) vector and PTEN protein-expression levels were determined by immunoblotting (**A**). SMC were incubated in basal medium (**B**) and in basal medium supplemented with H_2_O_2_ (**C**) and the relative number of apoptotic cells was evaluated following TUNEL-staining (expressed as % of total cells; *P<0.001; n = 6). HcASMC were transfected as follows: non transfected (NT); transfected with an empty plasmid without PTEN-cDNA (pControl); transfected with a plasmid coding for PTEN (pPTEN).

**Figure 4:**
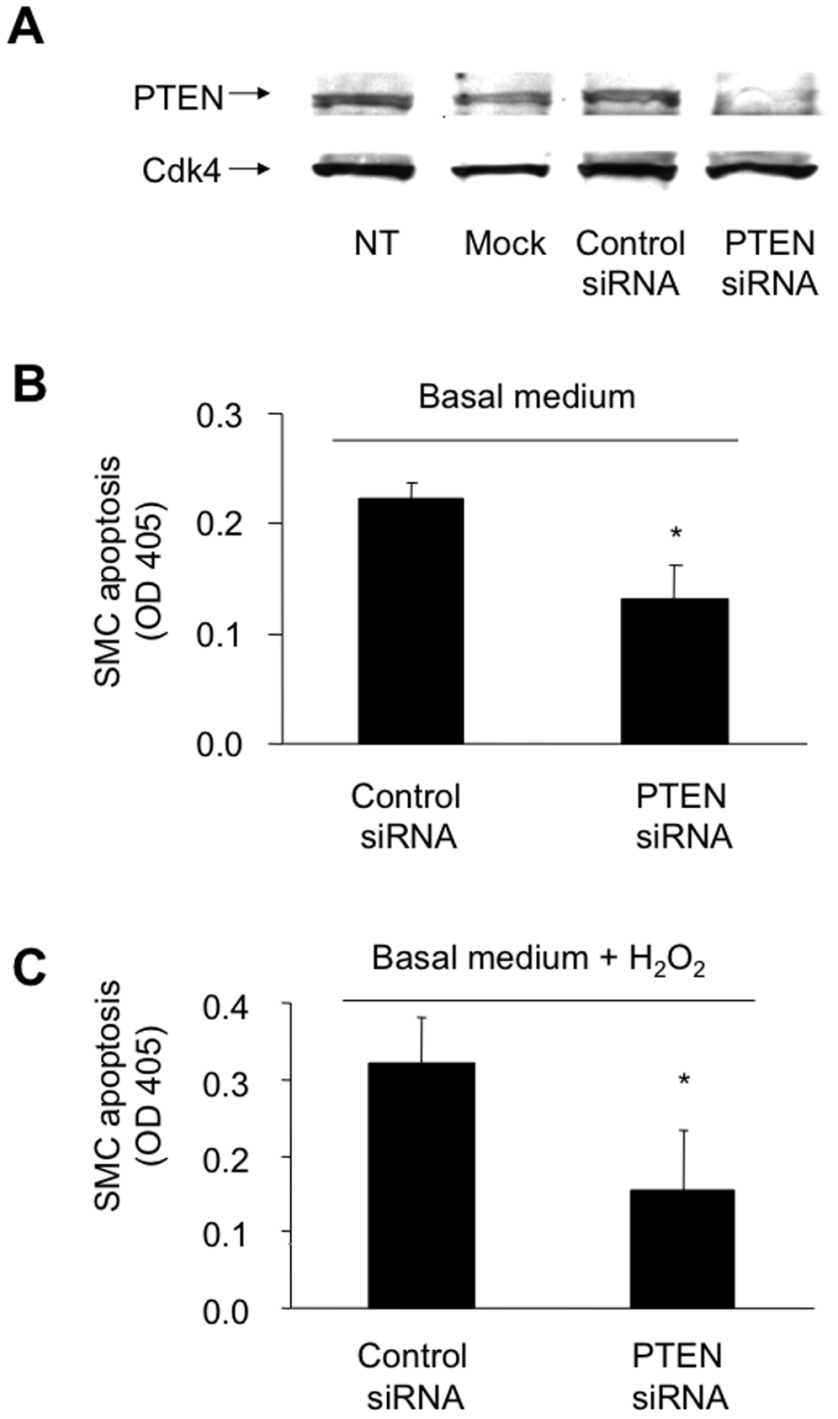
PTEN knock down attenuates SMC apoptosis under basal conditions or oxidative stress. **A**, PTEN expression following siRNA-mediated knock down. Protein expression was determined by western blotting using specific antibodies. Detection of Cdk4 served as a loading control. SMC were transfected as follows: non transfected (NT); mock transfected (without siRNA, but with lipid carrier); transfected with a non-targeting (scrambled) siRNA (Control siRNA); transfected with a targeting siRNA against PTEN (PTEN siRNA). SMC transfected with siRNA targeting PTEN or a scrambled control were incubated in basal medium in the absence (**B**) or presence of H_2_O_2_ (**C**). SMC apoptosis is expressed as mean OD405 ± SD using a cell death detection ELISA (*P<0.05; n = 3).

In the following experiments we investigated whether the observed PTEN-upregulation after vascular injury *in vivo* and after H_2_O_2_ stimulation in SMC *in vitro* influences apoptosis of SMC. We overexpressed PTEN by transfection of a plasmid coding for the WT form of PTEN in SMC and determined the number of apoptotic cells employing a, TUNEL assay. Our results clearly show that PTEN overexpression augments basal and H_2_O_2_-induced apoptosis as compared to cells transfected with an empty control vector (19.8%±4.4 vs. 5.6%±2.3; *P<0.001; Fig. 3A, and 44.5%±7.8 vs 25.5%±4.9; *P<0.001; Fig. 3B). On the other hand, we performed PTEN knock down experiments using specific siRNA targeting PTEN or scrambled control siRNA. Downregulation of PTEN protein expression by siRNA knock down was confirmed by immunoblotting of PTEN (Fig. 4A). Complementary, transfection with the scrambled siRNA control, MOCK transfection (only transfection reagent without siRNA) or no transfection clearly show an unaltered PTEN protein expression. Detection of Cdk4 served as a loading control. Following the knock down of PTEN, basal as well as H_2_O_2_-induced apoptosis of SMC were significantly attenuated (OD 0.22±0.014 vs. 0.13±0.03; *P<0.05; Fig. 4B, and 0.32±0.059 vs. 0.15±0.07; *P<0.05; Fig. 4C).

### Prevention of Akt-activation mediates the pro-apoptotic effect of PTEN

**Figure 5:**
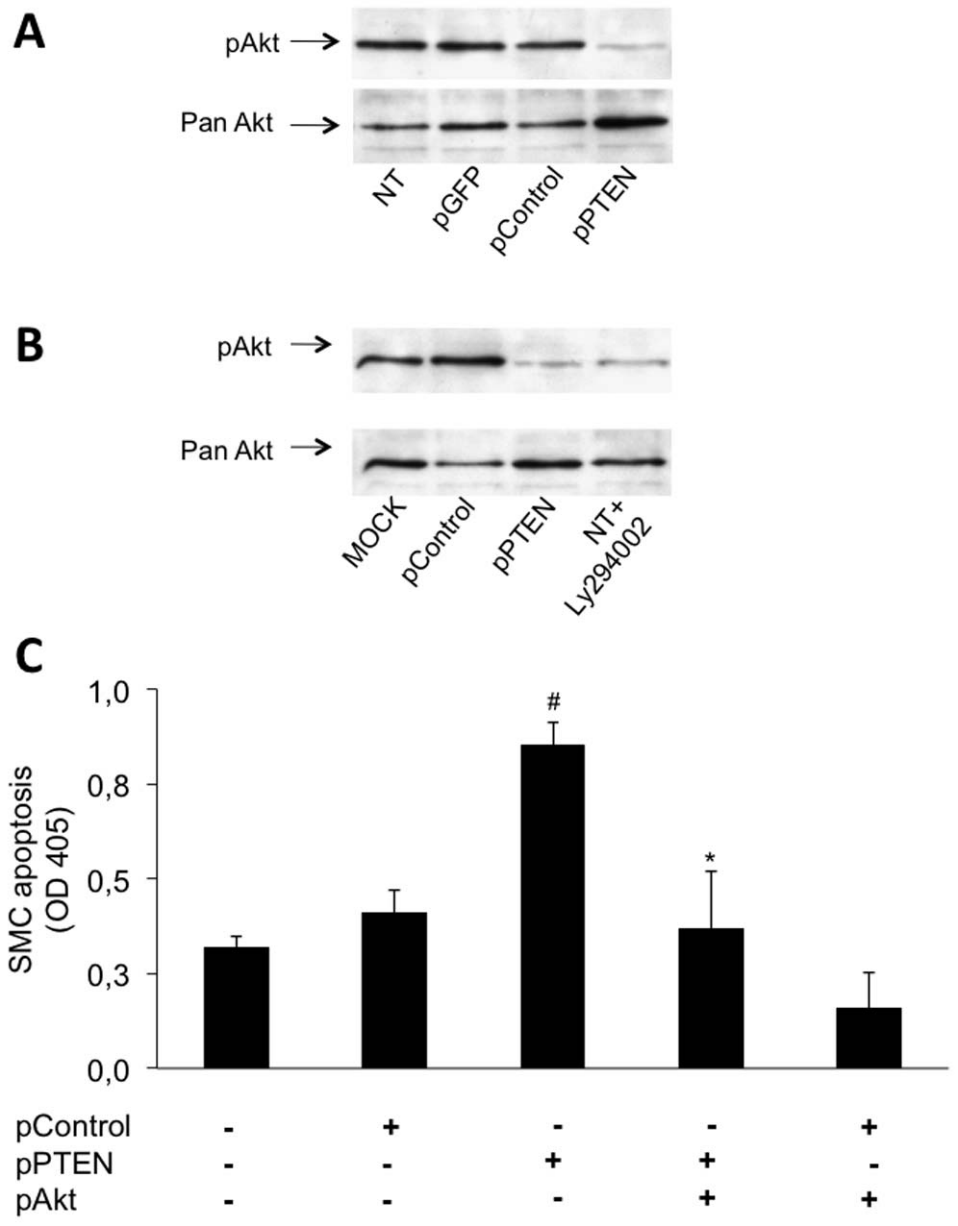
PTEN overexpression prevents serum-induced Akt phosphorylation after 15 min (A) and 30 min (B) serum induction using 10% FCS as determined by western blotting using an anti-pAkt-antibody. An antibody against total Akt was used as control. The potent PI3-K-inhibitor Ly294002 (50 nM) was supplemented to the medium for not-transfected cells. HcASMC were transfected as follows: non transfected (NT); transfected with a plasmid coding for GFP (pControl); transfected with an empty plasmid without PTEN-cDNA (pControl); transfected with a plasmid coding for PTEN (pPTEN); mock transfected (without plasmid, but with transfection reagent). PTEN- and Akt co-overexpression reverses PTENs pro-apoptotic effect (**C**). HcASMC were co-transfected with a WT form of PTEN and a constitutively active form of Akt. An empty plasmid was used as control. Following the magnetic separation of positively transfected SMC, apoptosis was determined after cell growth for 24 h and expressed as mean OD405 ± SD using a cell death detection ELISA (^#^ *P*<0.001, *P<0.001; n = 4).

Akt is a downstream regulator of the PTEN/PI3K-pathway and well known for its anti-apoptotic effects in several cell types [14]. To study the underlying mechanisms of PTENs pro-apoptotic effects, we determined the effect of PTEN overexpression vs. knock down on Akt-phosphorylation. As determined by immunoblotting using a specific antibody for phosphorylated (activated) Akt, overexpression of PTEN prevents the serum-induced phosphorylation of Akt (Fig. 5A) just as potent as a pharmacological PI3K-inhibitor (LY294002; 50 µmol/l; Fig. 5B). Representative controls of non-transfected cells, MOCK (only transfection reagent without plasmid), empty vector or a plasmid carrying GFP without PTEN clearly show Akt phosphorylation.

To assess, whether an impaired Akt-phosphorylation is responsible for the pro-apoptotic effect of an enhanced PTEN expression, we rescued the downstream Akt-signaling by overexpressing a constitutive active Akt mutant and determined the effect of PTEN-overexpression on SMC apoptosis. Overexpression of a constitutive active Akt mutant completely prevented the increased apoptosis in SMC overexpressing PTEN (OD 0.85±0.062 vs. 0.36±0.15; *P<0.001; Fig. 5C). Thus, these data indicate that an impairment of Akt-activation is indeed responsible for the pro-apoptotic effect of an increased PTEN expression.

### PTEN prevents SMC proliferation

**Figure 6:**
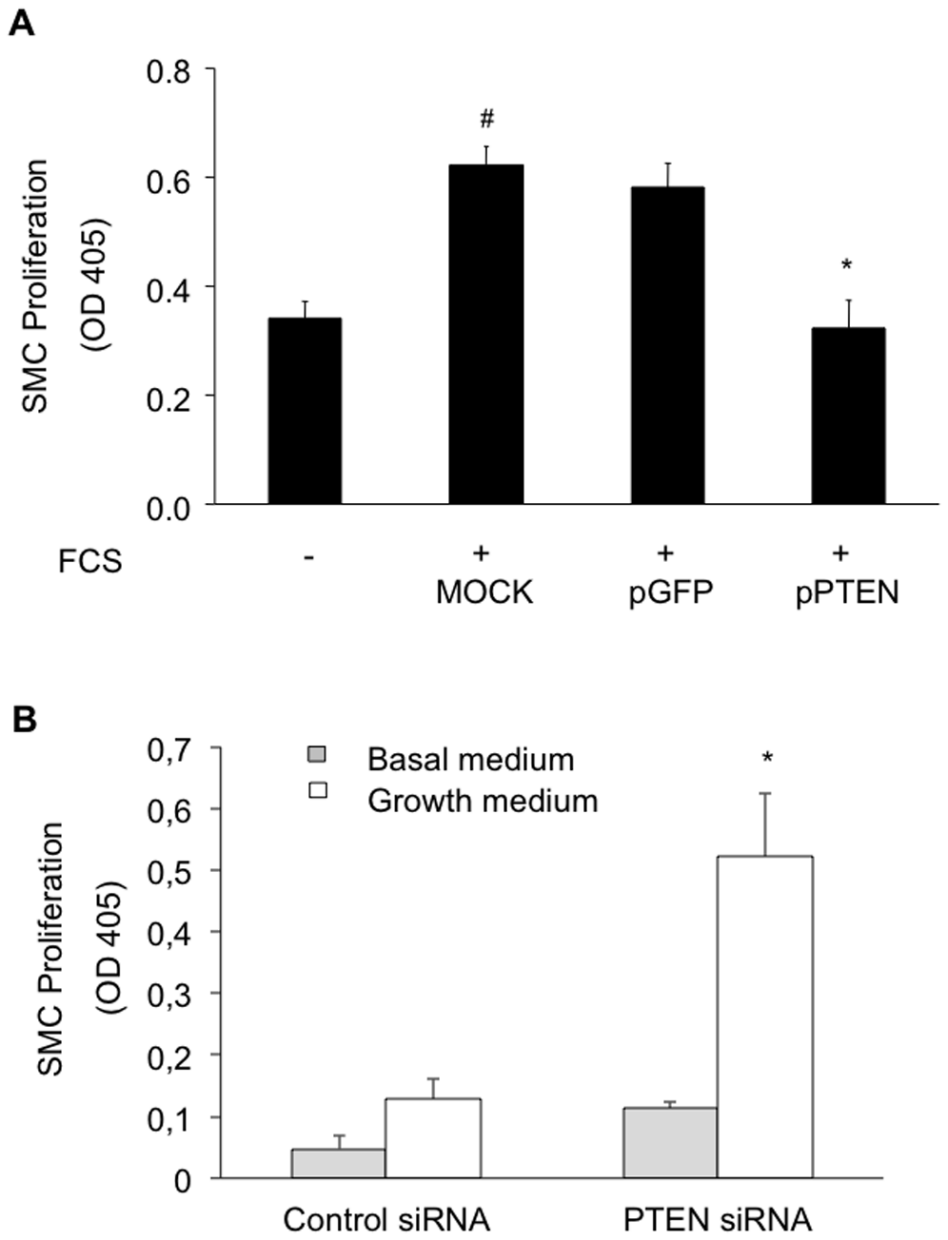
PTEN overexpression attenuates serum induced HcASMC proliferation (A). HcASMC transfected with a plasmid carrying WT-PTEN or a control vector carrying GFP alone were incubated either in basal medium or growth medium in the presence of BrdU. PTEN knock down enhances serum induced HcASMC proliferation (B). Cells were transfected with siRNA targeting PTEN or a scrambled control. Proliferation is expressed as mean OD450 ± SD as determined by anti-BrdU ELISA (^#^ *P*<0.001, *P<0.001; n = 4). HcASMC were transfected as following: mock transfected (without plasmid, but with transfection reagent); transfected with a plasmid coding for GFP (pGFP); transfected with a plasmid coding for PTEN (pPTEN); transfected with a non-targeting (scrambled) siRNA (Control siRNA); transfected with a targeting siRNA against PTEN (PTEN siRNA).

Considering that Akt is negatively regulated by PTEN and essential not only for prevention of apoptosis but also for growth factor-induced cell proliferation, we assessed the serum-induced SMC proliferation following the overexpression of PTEN. Indeed, serum-induced cell growth was significantly reduced in PTEN overexpressing cells as compared to cells transfected with a control plasmid only or MOCK transfected cells (OD 0.58±0.045 vs. 0.32±0.051; *P<0.001; Fig. 6A). In contrast, siRNA-mediated knock down of PTEN significantly enhanced SMC proliferation in quiescent as well as serum-stimulated SMC (OD 0.048±0.0204 vs. 0.128±0.009 and 0.114±0.033 vs. 0.5225±0.103; *P<0.001; Fig. 6B).

## Discussion

Intimal hyperplasia following vessel injury and angioplasty is caused by exceeding healing processes, which correlate with the extent of the initial injury. However, the mechanisms inducing and augmenting the initial apoptosis of SMC following injury have not been entirely elucidated yet. Hence, identification of the key mechanisms regulating VSMC function will help to understand cellular responses to vascular injury.

In the current study, we provide evidence that PTEN is a critical regulator of early apoptotic cell death of SMC after vascular injury. We demonstrate for the first time that PTEN expression is up regulated after carotid artery injury *in vivo*. *In vitro* analyses indicate that this up regulation is triggered by an H_2_O_2_-dependent process rather than by mitogenic stimuli or strain stress. Furthermore, we show that increased PTEN levels augment apoptosis of SMC by inhibiting the activation of Akt, thereby interfering with its downstream anti-apoptotic effects. Consistently, the proliferation of SMC was significantly decreased after PTEN overexpression, while the knock down of PTEN resulted in a significant increase of SMC proliferation.

We initially focused on PTEN's expression in the early phase after vessel dilatation *in vivo*. Following vascular injury, we observed a robust upregulation of PTEN expression in medial SMC. The increased PTEN expression was accompanied by an augmented apoptotic cell death rate of SMC as previously described by other studies [15]. We could further show that PTEN is up regulated and SMC apoptosis is augmented following the exposure of SMC to H_2_O_2_ *in vitro*. This finding is in accordance with the results of a recent study reporting that oxidative stress caused by exogenous peroxynitrite significantly increased PTEN-levels in pancreatic *β*-cells which resulted in an augmented apoptotic cellular response [16]. Likewise, studies using human umbilical vein endothelial cells (HUVECs) demonstrated, that PTEN expression increases after peroxynitrite treatment and that this leads to an augmented apoptotic response[17]. In the same report, *in vivo* experiments on aortic rings of diabetic rats showed a robust PTEN up regulation following hyperglycemia-driven peroxynitrite generation and a concomitant increase of apoptotic cells in the endothelium. Mechanistically, it has been observed that oxidative stress triggers the nuclear accumulation of APE1/Ref-1, a prominent redox regulator in eukaryotic cells. This is accompanied by activation of Egr-1, which transactivates PTEN transcription and upregulates PTEN protein levels [18]. Indeed, Egr-1 was found to be activated early after vascular injury[19]. Thus, following vascular injury, in the presence of enhanced ROS levels, the transcription factor Egr-1 transactivates PTEN expression in an APE1/Ref-1-dependent manner, thereby mediating cellular responses to oxidative stress, which results in SMC apoptosis[20].

ROS-induced oxidation is another important mechanism controlling PTEN activity. PTEN oxidation occurs in pathological situations characterized by chronic oxidative stress, e.g. in diabetes, which leads to persistent inactivation of PTEN [21]. Particularly, low levels of endogenous ROS have been demonstrated to prevent PTEN activation[22]. In contrast, other reports indicate, that high levels of endogenous or exogenous ROS rather increase PTEN-expression and activity [23], [24]. Given these discrepant reports, we aimed to determine the impact of PTEN oxidation in vivo following vascular injury. However, PTEN oxidation was not found to be enhanced at 12 h following balloon-induced vascular injury, indicating that PTEN oxidation does not attenuate PTEN activity under these conditions.

Our data provide evidence that the pro-apoptotic effect of PTEN is due to the inhibition of Akt activation. Accordingly, PTEN null cells exhibit higher Akt kinase activity and are more resistant to apoptosis (reviewed in [25]). According to several studies, Akt is activated after vessel injury by a variety of stress-inducing factors resulting in an attenuation of apoptotic cell death[10], [26]. Morever, insulin is a potent inducer of Akt-phosphorylation. Thus, while being potentially protective during the first hours after vascular injury, hyperinsulinemia will contribute to VSMC-hyperproliferation and increased neointima formation.

Furthermore, our data indicate that PTEN upregulation prevents cell proliferation showing that PTEN negatively regulates Akt activation and thus VSMC proliferation which is required for hyperplasia and restenosis. Likewise, in previous reports employing PTEN overexpression, an inhibition of proliferation and migration of VSMCs was demonstrated [5]. More recently, i*n vivo* experiments substantiated that adenoviral-mediated overexpression of PTEN in a rat carotid injury model inhibits neointimal hyperplasia through induction of apoptosis and inhibition of cell proliferation [6], [27]. In contrast, SMC-specific PTEN-deficient mutant mice exhibited enhanced medial and intimal SMC hyperplasia, and an increased production of a family of pro-inflammatory cytokines and chemokines like SDF-1α, which were up regulated in a NF-kB-dependent manner [28], [29] In a more recent study, Nemenoff et al. investigated the direct *in vivo* effects of PTEN inactivation selectively in VSMC. elevated SDF-1α levels produced by SMC following PTEN inactivation enhanced the recruitment of inflammatory cells and enhanced neointima formation [8].

Concluding, upregulation of PTEN after vascular injury resulted in enhanced apoptosis of VSMC, which augments cell loss in the vessel wall in the early phase after angioplasty. This may be relevant since we previously demonstrated that early trauma-induced SMC apoptosis results in increased late neointima formation [10]. At later time points, apoptosis seems to be confined to SMC of the developing neointima and, therefore, may be beneficial by limiting lesion growth [30], [31], [32]. However, PTEN-activation by therapeutic means should not follow instantly after vascular dilatation but should be applied at later time points to prevent initial exacerbation of apoptosis-induced vessel injury. Our data further add to the understanding of PTEN-dependent mechanisms in the initial phase following vascular injury and may have novel implications for the future design of therapeutic interventions to prevent restenosis.

## Supporting Information

Figure S1 **A**, H&E-stained cross sections of an uninjured and an injured carotid artery 12 h after balloon injury are shown. In the injured artery, strongly damaged areas can be identified by the lack of nuclei (blue). **B**, Immunoreactivity of aSMA (green), PTEN (red) and nuclei (DAPI, blue) is shown in representative sections of an injured rat carotid artery 12 h after balloon injury. Immunoreactivity of PTEN (green), apoptotic nuclei (TUNEL, red) and of nuclei (blue) is shown in undilated vessels. **C**, Representative pictures showing the squared area of a whole vessel cross section (lower panel) in 4× magnification. The lesions can be distinguished from the intact vessel area due to the reduction of DAPI-stained nuclei and the TUNEL+ nuclei in the severely damaged region.
(PDF)

Figure S2 **A**, PTEN-expression is upregulated following incubation of VSMC with H_2_O_2_ for 12 h. The simultaneous incubation with catalase but not with inactivated catalase prevented the H_2_O_2_-induced upregulation of PTEN protein levels. **B**, Following 20 min of H_2_O_2_ incubation (500 nM), almost all of the cellular PTEN protein- content is oxidized as indicated by immunoblotting and a band shift seen in non-reducing gels. In contrast, incubation of VSMC with H_2_O_2_ for 12 h increases total PTEN protein content but does not result in a prominent oxidation (band shift) of the protein. PTEN expression in dilated arteries is robustly increased at 12 h following injury, but no significant oxidation of the protein (no band shift) is detected by immunoblotting under non-reducing conditions.
(PDF)
