# Supplementary figures and images for: Role of the Phosphatase PTEN in Early Vascular Remodeling
Source: PLoS One. 2013 Mar 22;8(3):e55445. doi: 10.1371/journal.pone.0055445 (PMC3606387; doi:10.1371/journal.pone.0055445)

# Supplemental online figure 1

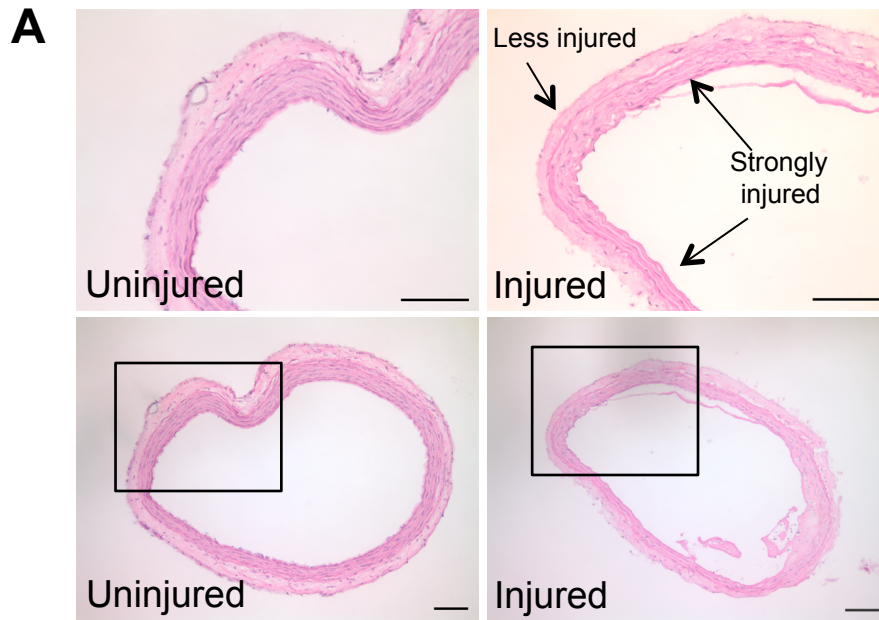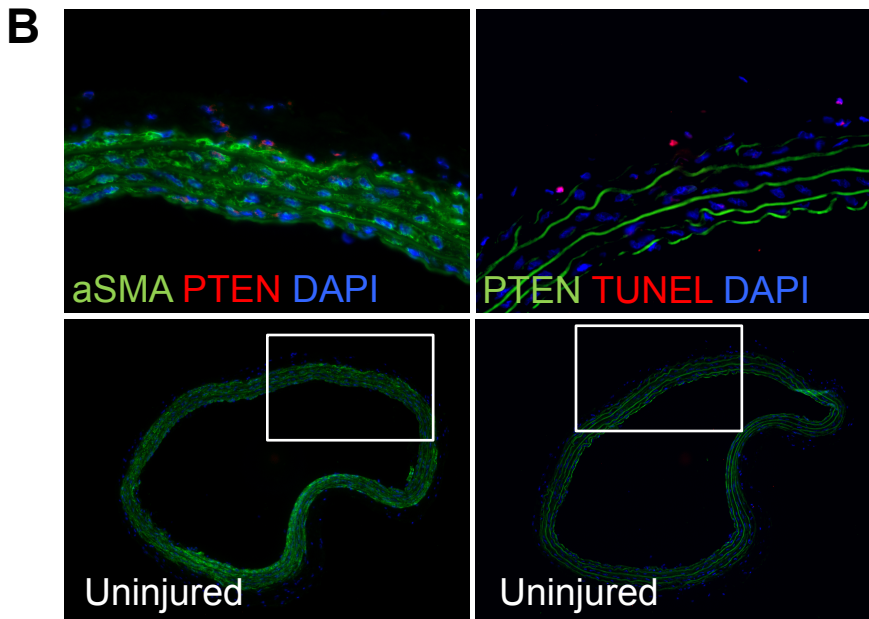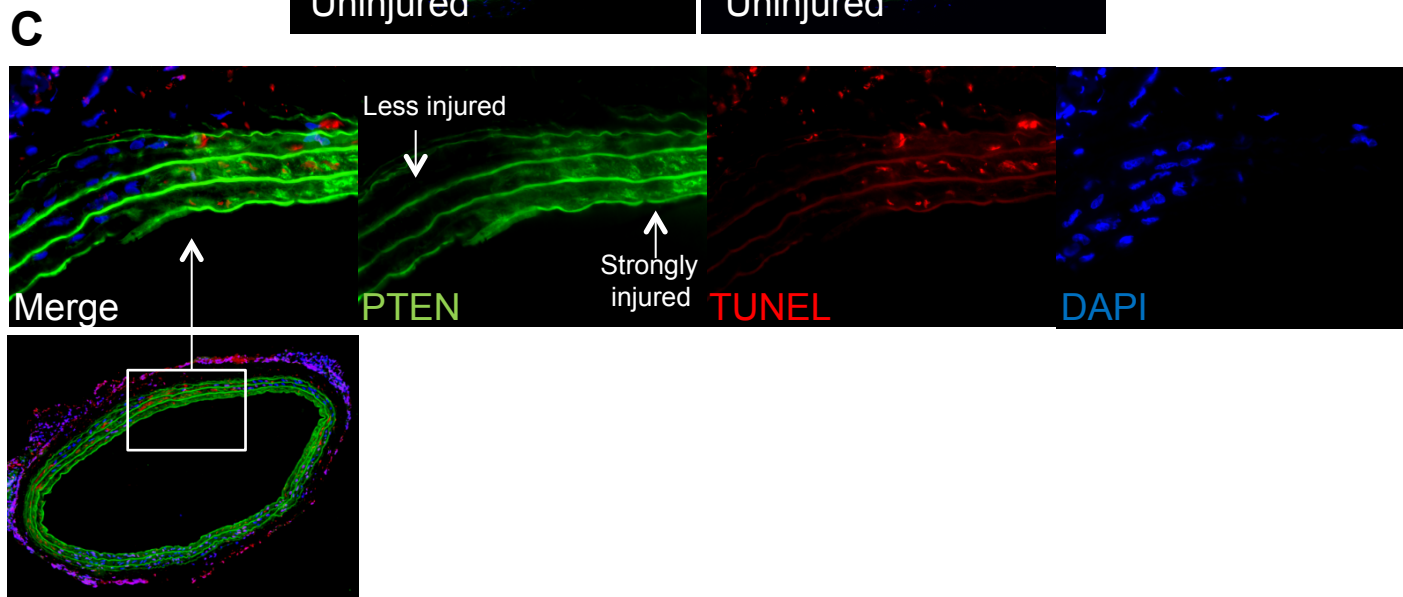

Supplement: Figure S1 — A, H&E-stained cross sections of an uninjured and an injured carotid artery 12 h after balloon injury are shown. In the injured artery, strongly damaged areas can be identified by the lack of nuclei (blue). B, Immunoreactivity of aSMA (green), PTEN (red) and nuclei (DAPI, blue) is shown in representative sections of an injured rat carotid artery 12 h after balloon injury. Immunoreactivity of PTEN (green), apoptotic nuclei (TUNEL, red) and of nuclei (blue) is shown in undilated vessels. C, Representative pictures showing the squared area of a whole vessel cross section (lower panel) in 4× magnification. The lesions can be distinguished from the intact vessel area due to the reduction of DAPI-stained nuclei and the TUNEL+ nuclei in the severely damaged region. (PDF) [file pone.0055445.s001.pdf]

**A**

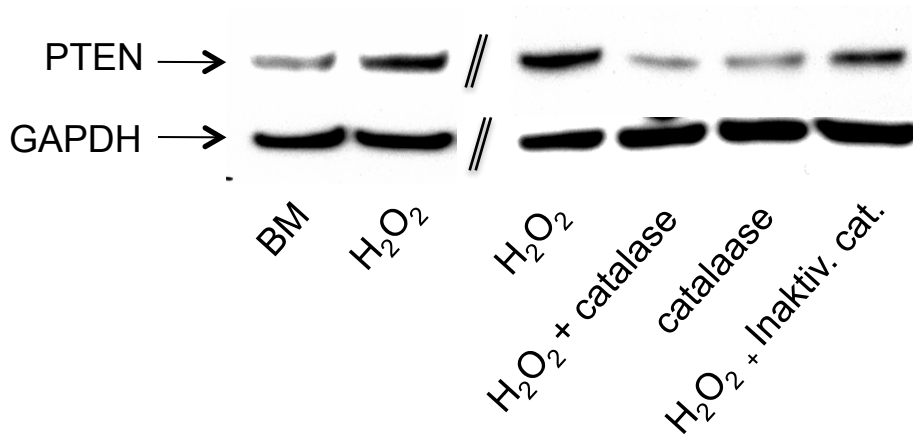

**B**

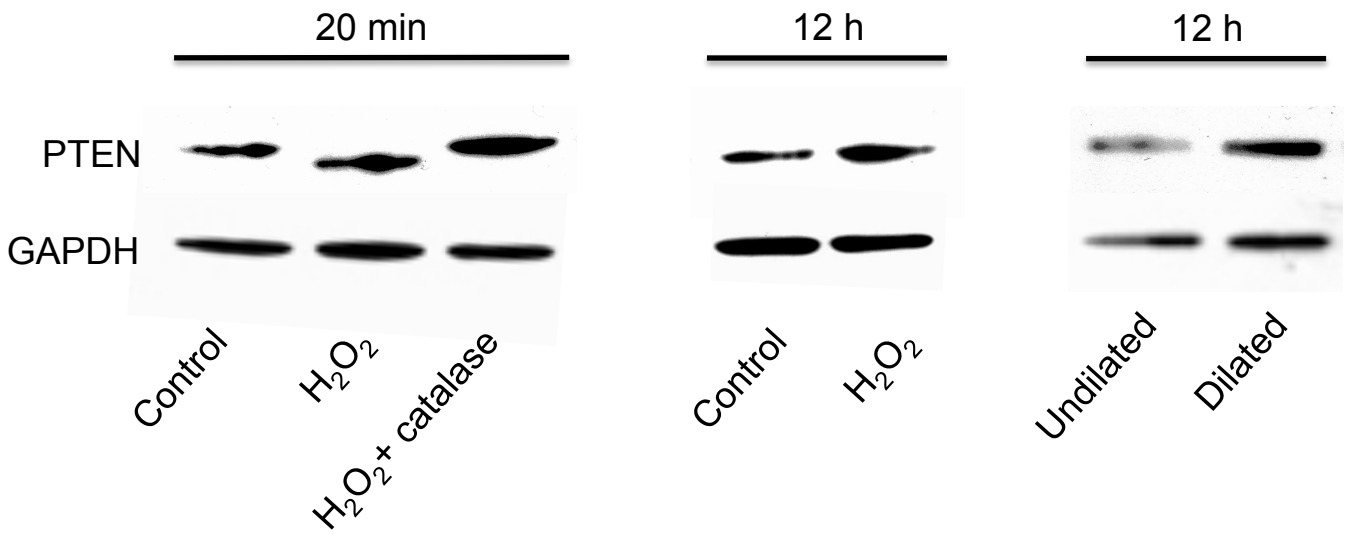

Supplement: Figure S2 — A, PTEN-expression is upregulated following incubation of VSMC with H2O2 for 12 h. The simultaneous incubation with catalase but not with inactivated catalase prevented the H2O2-induced upregulation of PTEN protein levels. B, Following 20 min of H2O2 incubation (500 nM), almost all of the cellular PTEN protein- content is oxidized as indicated by immunoblotting and a band shift seen in non-reducing gels. In contrast, incubation of VSMC with H2O2 for 12 h increases total PTEN protein content but does not result in a prominent oxidation (band shift) of the protein. PTEN expression in dilated arteries is robustly increased at 12 h following injury, but no significant oxidation of the protein (no band shift) is detected by immunoblotting under non-reducing conditions. (PDF) [file pone.0055445.s002.pdf]
